# From prediction to control: Age-related dissociations between implicit regularity encoding and attentional modulation during perception–action integration

**DOI:** 10.1016/j.dcn.2026.101739

**Published:** 2026-05-09

**Authors:** Roula Jamous, Katharina Graf, Leonore Luise Ilgenstein, Christian Frings, Christian Beste

**Affiliations:** a Cognitive Neurophysiology, Department of Child and Adolescent Psychiatry, Faculty of Medicine, TU Dresden, Schubertstrasse 42, Dresden 03107, Germany; b General Psychology, Department of Psychology, University of Trier, Trier, Germany; c German Center for Child and Adolescent Health (DZKJ), partner site Leipzig/Dresden, Dresden, Germany

**Keywords:** Perception action integration, Predictive coding, Adolescence, Adults, EEG, Alpha, Theta

## Abstract

Adolescence is a key period for the maturation of cognitive control, yet it remains unclear how developing brains transform learned regularities into flexible, goal-directed behavior. This study examined whether (pre-)adolescents can exploit the predictability of task-irrelevant information to improve perception–action integration. Using a distractor–response binding paradigm with varying predictability and EEG recording, we compared behavioral and neural indices between (pre-)adolescents (N = 35) and adults (N = 33). Behaviorally, adults—but not (pre-)adolescents—benefited from high predictability, showing reduced binding costs without changes in binding benefits. Time–frequency analyses revealed that adults exhibited theta-band modulation after prime onset and alpha-band modulation around probe onset, consistent with binding and attentional shielding mechanisms. These oscillatory modulations were absent in (pre-)adolescents, suggesting reduced integration of predictability into event-file representations. However, temporal generalization multivariate pattern analysis (MVPA) revealed above-chance decoding of high versus low predictability in both groups, including pre-prime intervals. Thus, (pre-)adolescents represented probabilistic structure but failed to use it to modulate behavior or attentional control. This dissociation implies that while implicit encoding of environmental regularities is functionally mature, the top-down mechanisms needed to implement this knowledge proactively are still developing. These findings highlight a developmental shift from representation to regulation linking the maturation of alpha–theta coordination to the emergence of flexible action control during adolescence.

## 1 Introduction

Adolescence is a pivotal period for neurodevelopment marked by substantial behavioral refinement and neural reorganization (see Best and Ban, 2021; Johnson et al., 2016; Spear, 2013; Velthuis and McAlonan, 2022). These maturational processes support the gradual enhancement of action control, i.e., the ability to select, coordinate, and adjust actions in line with internal goals. Whether navigating social situations, managing school tasks, or performing routine activities, successful action control is essential for achieving goal-directed outcomes. While the foundations of action control emerge in early childhood, its successful implementation continues to mature into adulthood. Similarly, key structural features of the brain, such as overall size, cortical folding, and regional specialization, are largely established by early childhood (e.g., Giedd et al., 1996; Gilmore et al., 2018; Kelly et al., 2024; Lenroot and Giedd, 2006; Nie et al., 2013), while the functional organization of neural networks, especially in the frontal cortex, continues to evolve throughout adolescence (Caballero et al., 2016, Crone and Steinbeis, 2017, Johnson et al., 2016, Kolk and Rakic, 2022). Furthermore, processes such as synaptic pruning and myelination (Huttenlocher, 1990, Luna et al., 2004, Zeiss, 2021) contribute to more efficient intra- and inter-regional communication, thereby supporting the maturation of action control. This trajectory reflects not the emergence of entirely new abilities but the progressive refinement of existing functions, resulting in increased accuracy and consistency in goal-directed behavior.

A central aspect of action control is the integration of perception and action: the process by which individuals bind stimulus features, responses, and their effects into coherent, retrievable representations known as event-files. According to the theory of event coding (TEC) (see Hommel, 2009, Hommel, 2019; Hommel et al., 2001) and the binding and retrieval in action control (BRAC) framework (Beste et al., 2026, Frings et al., 2020, Frings et al., 2024a), re-encountering any element of a prior stimulus-response episode can trigger retrieval of the entire event file. While this facilitates efficient behavior under stable conditions, it can impair performance when task demands change, requiring reconfiguration or suppression of previously established bindings. Research suggests that children and adolescents exhibit immature perception-action integration, as evidenced by “stickier” stimulus-response bindings – reflected in larger partial repetition costs compared to adults (Hommel et al., 2011; Dilcher et al., 2021a). This indicates that young individuals may struggle more with flexibly updating or overriding prior event files. However, the mechanisms underlying these developmental differences remain unclear. Are these bindings genuinely stronger in younger individuals? Do they have greater difficulty reconfiguring event files, or is their retrieval less selective, leading to interference from irrelevant information?

One potential contributor to these difficulties is immature attentional control. Compared to adults, children and adolescents are more susceptible to distraction and have greater difficulty sidelining task-irrelevant information (e.g., Cowan et al., 2006; Hobbiss and Lavie, 2024; Huang-Pollock et al., 2002; Pritchard and Neumann, 2009). Such limitations may impair their ability to modulate the retrieval or updating of event files based on current task demands. Interestingly, previous studies showed that healthy adults thrive in suppressing task-irrelevant information during perception-action integration processes when the predictability of task-irrelevant features is clearly increased (Jamous et al., 2024, Schmalbrock et al., 2022). Predicting upcoming stimuli modulates perception-action integration processes as evidenced at behavioral and neurophysiological levels: It was found that predictability specifically reduces binding costs while binding benefits are unaffected. The neurophysiological results (Jamous et al., 2024) indicate that predictability is also integrated into the event-file as an “instruction” to ignore task-irrelevant features in the following event. In this case, a partially overlapping event-file may not be retrieved completely, because attention is withdrawn from task-irrelevant features which overlap. The consequence is that there is no need for event-file reconfiguration and thus no decline in performance. The concept that human brains engage in probabilistic computations that are continuously updated based on the actual evidence has been assumed to be relatively independent of age (Bubic et al., 2010, Clark, 2013, Darriba and Waszak, 2018, Mendonça et al., 2020, Philippsen and Nagai, 2022). Thus, the question arises whether children and adolescents can also engage compensatory mechanisms – such as utilizing predictability of task-irrelevant information – to support more efficient perception-action integration despite their attentional constraints. To fully understand how action control matures, it is essential to examine not only behavioral performance but also the underlying neurophysiological mechanisms.

Recent evidence indicates that interactions between neural frequency bands reveal a hierarchical organization of brain regions that collectively regulate action control and cognitive flexibility, aligning with the BRAC framework (Beste et al., 2023, Zhupa and Beste, 2026). Theta band activity (TBA) plays a pivotal role in cognitive control and memory processes by signaling unexpected changes in the environment, thereby supporting adaptive behavior (Cavanagh and Frank, 2014, Williams et al., 2021). Moreover, TBA facilitates the binding and retrieval of event-files, which represent integrated sensory, motor, and cognitive information (Beste et al., 2023, Harper et al., 2017, Pscherer et al., 2019, Wendiggensen et al., 2023). Beta band activity (BBA) is closely linked to the maintenance and temporal structuring of these event-files, determining how long previously encoded representations can influence subsequent behavior (Pastötter et al., 2021, Wendiggensen et al., 2022). Alpha band activity (ABA), as proposed by the “inhibition timing hypothesis,” serves as a key mechanism for neural inhibition and attentional modulation, influencing sensory processing, memory retrieval, and motor planning (Klimesch, 2012, Klimesch et al., 2007). Within the BRAC framework, ABA does not directly contribute to the formation of event-files but modulates their activation based on task relevance through top-down control over TBA (Beste et al., 2023, Prochnow et al., 2022a, Wendiggensen et al., 2023). This is in line with many findings suggesting that ABA reflects attentional processing (Foxe and Snyder, 2011, Herrmann and Knight, 2001, Klimesch, 2012). The improvement of healthy adults in suppressing task-irrelevant information during perception-action integration processes (high predictability) was linked to higher TBA during binding, and higher ABA during retrieval. The increase in ABA in highly predictable conditions during retrieval indicates a top-down attentional control mechanism, while the increase of TBA likely reflects that predictability is bound to the perceptual features of the stimuli (Beste et al., 2023, Jamous et al., 2025). While the inhibition‑timing hypothesis (Klimesch, 2012) emphasizes alpha‑driven suppression of task‑irrelevant processing, other theoretical accounts highlight its broader role in regulating cortical excitability and rhythmic coordination across perceptual and executive systems. In this view, alpha activity indexes cycles of functional inhibition and communication that shape when and where sensory representations are prioritized (Foster and Awh, 2019, Jensen and Mazaheri, 2010, Lenartowicz et al., 2025). Hence, ABA can flexibly gate information flow rather than solely inhibit it. Within the BRAC framework, this broader interpretation suggests that alpha contributes to attentional weighting and contextual updating of event files by modulating excitability in feature‑specific networks. Likewise, TBA, while linked to conflict monitoring and prediction‑error signaling (Cavanagh and Frank, 2014), also supports communication between distributed networks that maintain and update task rules (Cohen, 2014). Considering these complementary roles allows a more nuanced interpretation of how alpha–theta interactions underpin perception–action integration.

The present study investigates the development of perception–action integration across (pre-)adolescence, focusing on the flexibility of event-file updating and the potential for compensatory mechanisms to mitigate partial-overlap costs. Building on our previous study in healthy adults (Jamous et al., 2024), we employed an identical distractor–response binding paradigm with two levels of predictability while recording EEG. This design enables a direct comparison of behavioral and neurophysiological correlates between (pre-)adolescents and adults. We hypothesize that (pre-)adolescents do not benefit in the same way as adults from increased predictability of distractor features, indicating less efficient use of attentional control in event-file updating. At the neural level, we expect the oscillatory modulations found for adults in TBA and ABA to be weaker or temporally delayed in (pre-)adolescents—consistent with immature attentional regulation and cognitive control. To complement the oscillatory analyses, we applied multivariate pattern analysis (MVPA) (King and Dehaene, 2014) to capture distributed neural representations underlying perception–action integration. MVPA can detect fine-grained, spatially distributed information patterns that may not appear in traditional power-based analyses (Grootswagers et al., 2017, Hebart and Baker, 2018, Isik et al., 2014, King and Dehaene, 2014). This approach is particularly useful in developmental samples, where neural responses are more variable and less spatially localized (Somerville and Casey, 2010). MVPA thus reveals whether adolescents encode probabilistic regularities in representational formats comparable to adults—even when behavioral or oscillatory markers appear attenuated. Linking decoding accuracy with frequency-specific neural dynamics may further clarify how distributed coding and dynamic coupling jointly shape the maturation of flexible, goal-directed action control (Yu et al., 2023a, Yu et al., 2023b). Given that adolescents can acquire implicit probabilistic regularities similarly to adults (e.g., Amso and Davidow, 2012; Lukasova et al., 2018; Meulemans et al., 1998; Reber, 2013; Vinter and Perruchet, 2000), we anticipate comparable MVPA classification performance between groups, suggesting that the ability to encode and utilize predictability is already functionally mature. Together, these behavioral and neural indices delineate how predictability and attentional control contribute to the developmental refinement of perception–action integration.

## 2 Material and methods

To ensure comparability of the results, analyses were conducted in accordance with the methods applied in our previous paper (Jamous et al., 2024), including the focus on trials with response change and distractor repetition (so-called RCDR trials) for the neurophysiological analysis (details see below).

### 2.1 Samples

We assessed a total of *N* = 38 (pre-)adolescent participants aged 11–17 years without any neurological or psychiatric disorders. Participants were recruited as a convenience sample via advertisements and an in-house database. After exclusions due to excessive artefacts in the EEG data (*N* = 4) or behavioral performance significantly below average (*N* = 3), the final sample consisted of *N* = 35 subjects (23 females, *M*_*Age*_ = 13.7, *SD*_*Age*_ = 1.5). Specifically, participants were excluded when overall task accuracy fell more than 3 standard deviations below the group mean or below chance‑level performance (< 50% correct), ensuring that analyses were based on participants who reliably understood and performed the task. The task was conducted in one session with EEG recording and took participants 30 min, including practice trials and both predictability conditions. All participants and their legal guardians reported the absence of psychiatric or neurological disorders and normal or corrected-to-normal vision during a brief telephone screening. Before the study procedures started, participants and their legal guardians provided written informed consent. The study was approved by the ethical committee of the Technical University Dresden. To clearly classify the results of this study, an adult control group is required. For this purpose, the healthy adult sample of *N* = 33 from one of our previous papers (Jamous et al., 2024) was used (17 females, *M*_*Age*_ = 25.7, *SD*_*Age*_ = 3.6). Schmalbrock et al. (2022) showed that for the used paradigm, a minimum of *N* = 27 participants is needed to detect medium-sized effects. Hence, each group’s sample size at hand should be reasonably powered to observe effects.

### 2.2 Task

**Fig. 1:**
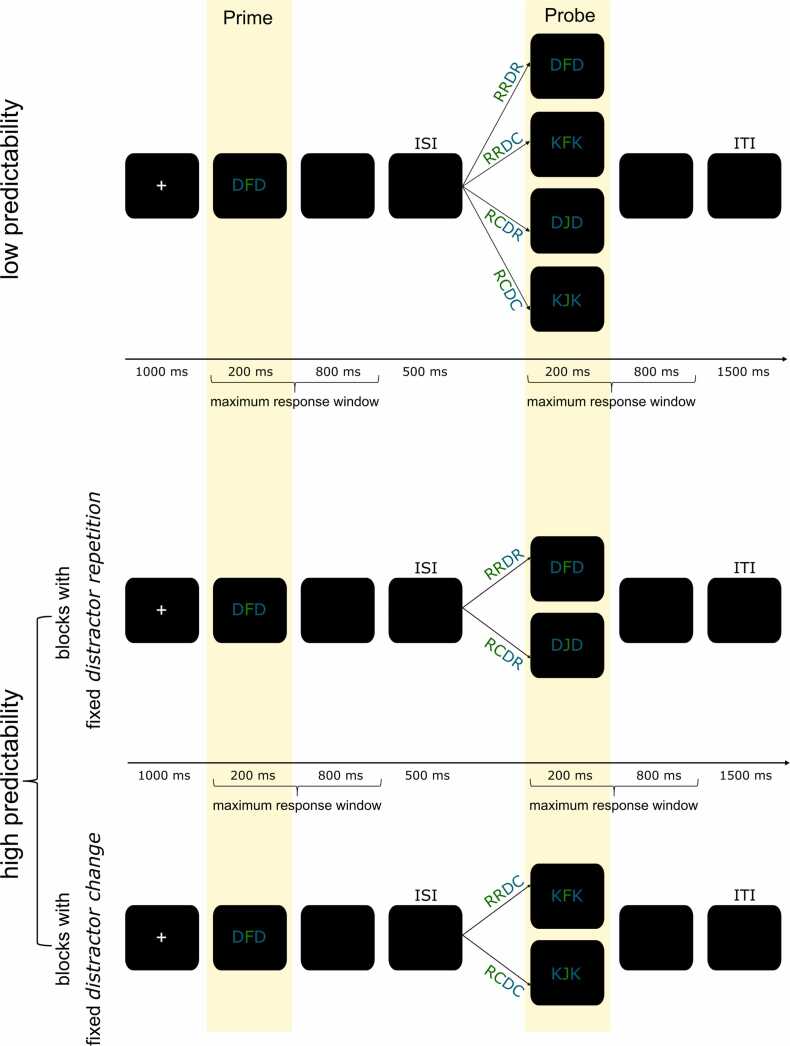
Schematic illustration of the distractor-response binding paradigm including two degrees of predictability (within-subject). The modified distractor-response binding paradigm, as already used in previous work in healthy adults was also used in this study (Jamous et al., 2024, Schmalbrock et al., 2022).

Participants performed the distractor-response binding paradigm with two levels of predictability (Hao et al., 2025, Jamous et al., 2024, Schmalbrock et al., 2022). Please refer to Fig. 1 for a depiction of the structure, timing, and manipulation of predictability.

The stimuli, event timing, and trial types, as well as the predictability manipulation, are depicted in Fig. 1. This task uses a prime-probe design; thus, each trial consists of a first stimulus (the prime) and a second stimulus (the probe), and participants must respond to each of them immediately. This design has been shown to reflect the binding and retrieval processes of event-file binding separately (Frings et al., 2020). While perceiving the prime, features of stimuli and (planned) responses are integrated into an event-file. During the probe, if at least one prime feature is repeated, this event-file will be retrieved again (Hommel, 2004). Depending on the degree of feature overlap between the prime and probe, the response to the probe is either facilitated (benefit), or the event file must be re-configurated, thus, the response to the probe is aggravated (cost; Frings et al., 2024b; Henson et al., 2014; Hommel, 1998; Prochnow et al., 2022b). Thus, only the probe response is examined on a behavioral level as it reflects retrieval of the prime (through benefit and cost), while indicating that the prime features were integrated in the first place (i.e., binding). On a neurophysiological level, activity around the prime and around the probe is observed separately to investigate both binding and retrieval individually.

In the distractor-response binding paradigm, participants must identify the centrally presented, green target letter (F or J, both of which are presented equally often as prime and probe targets) from a string of three letters. The two flanking letters, presented in blue, serve as task-irrelevant distractors (D or K, both of which are presented equally often as prime and probe distractors). Thus, the relation between the prime and probe is defined by the "response relation" (same or different target letter) and the "distractor relation" (same or different distractor). The response can be the same for the prime and probe (response repetition, RR) or different (response change, RC). Accordingly, the prime distractor can be presented again in the probe (distractor repetition, DR) or changed (distractor change, DC). These combinations result in four different trial types: RRDR (complete overlap between the prime and probe features); RRDC and RCDR (partial overlap between the prime and probe features); and RCDC (no overlap between prime and probe features; Schmalbrock et al., 2022). These four trial types are presented equally frequently throughout the experiment.

According to Frings et al. (2007), task-irrelevant features, such as distractors, are integrated and retrieved in the same way as task-relevant features. Thus, repeating the prime distractor leads to retrieving the prime event file during the probe. The distractor repetition binding (DRB) effect is defined as the difference between the benefit and the cost (Frings et al., 2007, Pastötter et al., 2021, Schmalbrock et al., 2022).

#### 2.2.1 Task structure and timing

Each trial began with a fixation cross (+) presented at the center of the screen for 1000 ms. Next came the prime display, which lasted at least 200 ms with a response window of up to 1000 ms beginning at display onset. The prime ended either when a response was carried out or after 1000 ms. Then, a blank screen was presented for 500 ms (the inter-stimulus interval, or ISI). Next, the probe display was presented for at least 200 ms until the end of the 1000-ms response window relative to probe onset. Any response during the response window ended the presentation of the probe display. Each trial was separated from the next by a blank screen for 1500 ms (intertrial interval, ITI). For the entire experiment, participants were instructed to place their left index finger on the F key and their right index finger on the J key on the keyboard and to respond to the prime and probe as quickly as possible while maintaining high accuracy.

#### 2.2.2 Predictability manipulation

To investigate the effects of predictability on the DRB effect, two levels of predictability were created by the order in which trial types were presented. Each predictability condition consisted of 192 trials, with breaks after each 48-trial block. Timing and order of events within a trial were the same for low and high predictability. In the low predictability condition, all possible trial configurations (derived from response relation, distractor relation, target, and distractor identity) were presented randomly intermixed. In the high predictability condition, each of the four separate blocks (each 48 trials) was limited to one kind of distractor relation (either DC or DR) with a fixed prime distractor identity (either D or K). This led to the following block types: Distractor repetition with only D as distractor; Distractor repetition with only K as distractor; Distractor change with D as a prime distractor and K as probe distractor; Distractor change with K as a prime distractor and D as probe distractor. Each of the blocks consisted of 24 response repetition trials and 24 response change trials that were randomly intermixed. The order of the blocks was also randomly intermixed across subjects. In this fashion, it was ensured that predictability was exclusively associated with the distractors (Schmalbrock et al., 2022). To avoid order effects, the order of the two predictability conditions was balanced across participants, thus, half of the participants started with the low predictability condition, and the other half with the high predictability condition.

### 2.3 EEG recording and preprocessing

EEG signals were recorded using 60-channel elastic caps (EasyCap Inc.) with equidistant Ag/AgCl electrodes (the reference electrode was placed at FPz and the ground electrode at θ = 58, ϕ = 78). BrainAmp EEG amplifiers (Brain Products Inc.) were used for this recording. The electrode impedances were kept below 5 kΩ. After recording at a sampling rate of 500 Hz without an online filter, the data were down-sampled to 256 Hz offline, and a band-pass filter (0.5–40 Hz) was applied. The reference was changed to an average reference. Technical artifacts were eliminated based on visual inspection during manual preprocessing. Recurring artifacts (e.g., pulse artifacts and horizontal and vertical eye movements) were removed by independent component analysis (ICA, infomax algorithm). After baseline correction from −200 to 0 ms, automated artifact rejection excluded any remaining artifacts (i.e., amplitudes less than 0.5 µV within a 100 ms period or greater than ±150 µV) and designated them as bad intervals 200 ms before and after the event. Segments including these bad intervals were rejected for further data analysis. Finally, all missing and eliminated channels were interpolated using a spherical method. The neurophysiological data were then segmented for all trials with correct responses to the prime and probe. The prime- and probe-locked segments (-3500–3500 ms) were organized by trial type in both high and low predictability conditions. Based on the results within the adult sample (Jamous et al., 2024), the neurophysiological analysis steps were focused on the RCDR trials for a direct comparison between the (pre-)adolescents and adults.

### 2.4 Time-frequency decomposition

To compare oscillatory activity in different predictability conditions within and between groups, a time-frequency transformation with Morlet wavelets (Morlet parameter of 5) was performed. The analysis was conducted within the 1–30 Hz range in 1 Hz steps and averaged the power within each frequency band. Since comparisons were also made between groups, normalization was necessary. For this purpose, the time interval of 700–200 ms before the prime stimulus onset was used, i.e., power values were transformed into decibel change relative to the −700 to −200 ms pre‑prime baseline before averaging and before any group‑level comparisons. To identify electrodes and time windows with significant differences in theta (4–7 Hz) and alpha (8–12 Hz) band activity between high and low predictability conditions, as well as between groups, nonparametric, cluster-based permutation tests were performed using FieldTrip (Maris and Oostenveld, 2007). A cluster was defined as two consecutive time points or two adjacent EEG channels. The reference distribution of the permutation test was approximated using the Monte Carlo method by performing 1000 random draws. This number of draws is common in non-parametric EEG statistics (Maris and Oostenveld, 2007), providing a stable p-value estimate while keeping computational time feasible for the current dataset. A cluster was considered significant if the corresponding p value was less than.05.

### 2.5 MVPA

We aimed at examining how the classification performance, based on the oscillatory activity, into a high vs a low predictability RCDR trial differs between the two groups. Based on evidence that adolescents acquire implicit probabilistic regularities to a similar extent as adults, we expect comparable classification performance across groups, indicating that the neural mechanisms supporting the encoding and utilization of predictability are already functionally mature. To this end, two different classifications were investigated within each group for the time around each the prime and the probe stimulus onset: the classifier being 1) trained and tested on the time course of TBA and 2) trained and tested on the time course of ABA. With a time-interval of −1000–1000 ms around stimulus onset (prime or probe), the classifications allow us to investigate at what time high and low predictability RCDR trials can be clearly distinguished. For each of the classifications, we used the MVPA-light toolbox (Treder, 2020) and trained a binary classifier to discriminate between the high and the low predictability trials. In general, we adopted the default settings of the toolbox including the LDA classifier (Treder, 2020). Furthermore, we applied shrinkage regularization of γ = 0.2 to stabilize covariance estimates and minimize overfitting. Classification performance was evaluated with 10‑fold cross‑validation, repeated 5 times across participants, and averaged across folds. The classifier’s predictive performance was quantified by the area under the receiver‑operating‑characteristic curve (AUC) relative to a 50% chance level. The MVPA analyses were performed on each participant’s dataset with the time course in each electrode as a feature vector (i.e., as input). First, we performed an MVPA to investigate at which time points after stimulus presentation the classifier can discriminate between both conditions based on the pattern of the bandpass filtered sensory-level data across all electrodes with the filter being set to 1) 4–7 Hz (i.e., theta power) and 2) 8–12 Hz (i.e., alpha power). The results were displayed via the AUC. Additionally, a temporal generalization analysis was carried out with the same feature vector to obtain information on the temporal dynamics of the activation patterns. We performed cluster-based permutation tests (based on Wilcoxon tests with a threshold set at ***p*** = 0.05) to test at which time points the AUC differed significantly from the chance level of 50% correct classification. To approximate the reference distribution, 1000 random draws were used. The statistical values of the cluster-based permutation tests were computed as the sum of all Wilcoxon-values within the time points.

### 2.6 Statistical analysis

The behavioral data were analyzed in a similar manner as in our previous paper (Jamous et al., 2024), while also including a comparison to the adult sample of said paper: All behavioral analyses were conducted for the reaction times to the probe (RT_pro_) in trials with correct responses to prime and probe. Additionally, only RT_pro_ longer than 200 ms and shorter than 1.5 interquartile ranges over the third quartile of each person’s RT distribution (Tukey, 1977) were included. The distractor repetition benefit was calculated as RRDC minus RRDR, distractor repetition cost as RCDC minus RCDR, and distractor repetition binding (DRB) (Frings et al., 2007, Opitz et al., 2020) as the difference of the first two (benefit minus cost). The statistical analyses of the behavioral data were performed using SPSS.

For adults, it has been shown that high predictability does not only reduce the DRB effect, but in fact eliminates the distractor repetition cost in total, without affecting the distractor repetition benefit at all (Jamous et al., 2024). This influence is driven only by trials in which distractors are repeated but lead to a partial overlap, i.e., RCDR. Therefore, behavioral performances were compared using repeated-measures ANOVAs on three levels: the DRB effect itself, its sections (distractor repetition benefit and distractor repetition cost), and the trial types RCDR and RCDC. All three ANOVAs included the within-subject factor predictability (high vs low) and the between-subject factor group ((pre-)adolescents vs adults). When necessary, Bonferroni-corrected post-hoc tests are stated. Additionally, the Bayes factor as BF_10_ is reported for all statistical analyses, to quantify the evidence for the null hypothesis. In the Bayesian *t*-tests, the default Cauchy prior was used with a scale of 0.707. For interpretation of the reported Bayes factors, we used the categorical evaluation: A BF_10_ of 1 indicates no evidence for H1, up to 3 as anecdotal, up to 10 as moderate, up to 30 as strong, up to 100 as very strong, and above 100 as extreme evidence for the H1. In contrast, values beneath 1 and up to 0.33 would be read as anecdotal, up to 0.01 as moderate, up to 0.033 as strong, up to 0.001 as very strong, and smaller than 0.001 as extreme evidence for the H0.

## 3 Results

### 3.1 Behavioral data

**Fig. 2:**
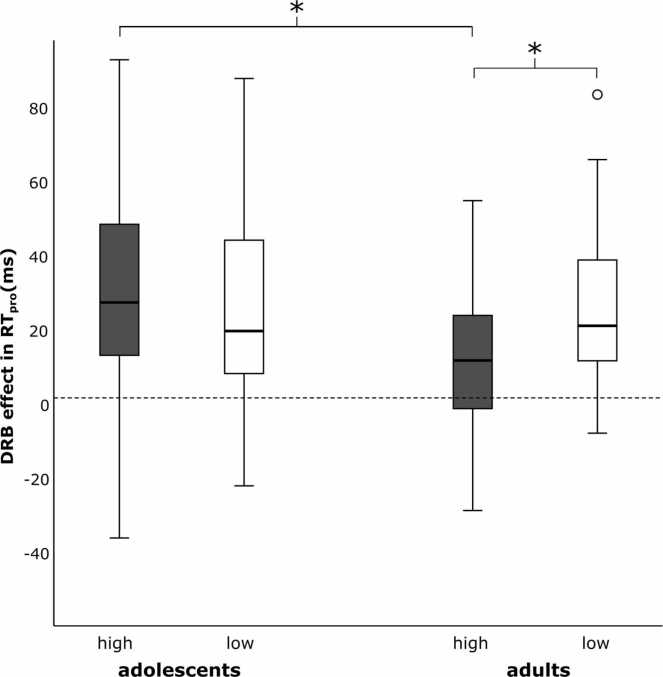
Comparisons of DRB effects in high and low predictability conditions between groups. DRB effects reflected by reaction times of the probe responses (RT_pro_ in ms). Boxes indicate inter-quartile range and median, whiskers extend to values up to 1.5 inter-quartile range beyond first and third quartile, respectively. Values outside this range are shown as individual points. *Significant differences at p < .05. **Significant differences at p < .001.

**Fig. 3:**
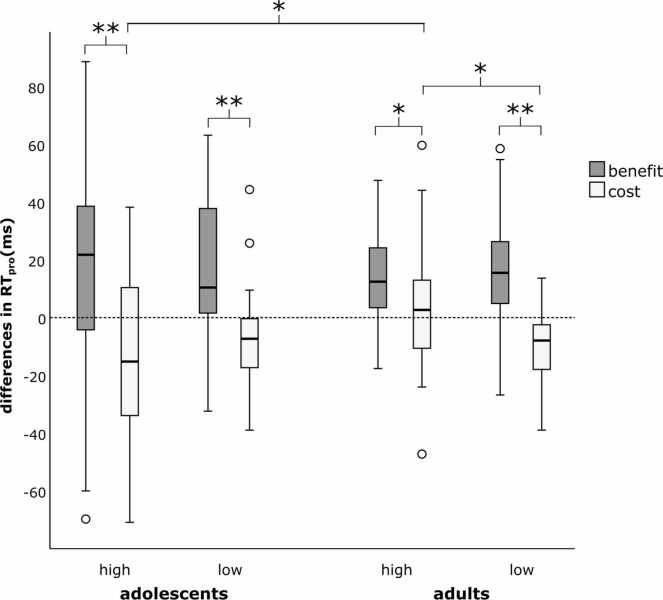
Comparison of DRB sections in high and low predictability conditions and groups. Distractor repetition benefit and distractor repetition cost reflected as differences in reaction times of the probe responses (RT_pro_ in ms). Distractor repetition benefit is calculated as RRDR minus RRDC, and distractor repetition cost as RCDR minus RCDC. Boxes indicate inter-quartile range and median, whiskers extend to values up to 1.5 inter-quartile range beyond first and third quartile, respectively. Values outside this range are shown as individual points. *Significant differences at *p* < .05. **Significant differences at *p* < .001.

The behavioral results for *N* = 35 (pre-)adolescent and *N* = 33 adult participants are depicted in Fig. 2 (overall DRB effect) and Fig. 3 (DRB sections). Regarding the overall DRB effect in RT_pro_ (see Fig. 2), a significant interaction of predictability * group was found (*F*(1,66) = 5.493, *p* = .022, *η*_*p*_^*2*^ = 0.077, *BF*_*10*_ > 100): according to Bonferroni-corrected post-hoc tests, (pre-)adolescents and adults did not differ in the low predictability condition (*M*_*Diff*_ = 0.52 ± 6.28, *p* = .935, *BF*_*10*_ = 0.25), but in the high predictability condition (*M*_*Diff*_ = 21.42 ± 7.03, *p* = .003, *BF*_*10*_ = 11.36). While adults showed a significantly smaller DRB effect in the high (11.24 ± 5.04) than in the low predictability condition (25.67 ± 4.51, *p* = .027, *BF*_*10*_ *=* 23.58), the DRB effect within (pre-)adolescents was not affected at all by predictability (*M*_*Diff*_ = 6.48 ± 6.22, *p* = .301, *BF*_*10*_ *=* 0.18).

The second ANOVA (see Fig. 3) revealed a significant three-way interaction of DRB section*predictability*group (*F*(1,66) = 5.493, *p* = .022, *η*_*p*_^*2*^ = .077, *BF*_*10*_ *=* 67.34). Bonferroni-corrected post-hoc tests revealed that all participants showed positive values for the distractor repetition benefit, which did not differ between predictability conditions (*M*_*Diff*_ = 0.95 ± 4.92, *p* = .847, *BF*_*10*_ *=* −12.83) nor groups (*M*_*Diff*_ = 5.59 ± 4.69, *p* = .238, *BF*_*10*_ *=* 1.17). For adults, only the low predictability condition led to a distractor repetition cost, i.e., a negative value (-10.75 ± 2.62), while in the high predictability condition, the cost was significantly decreased and basically eliminated (2.10 ± 4.33, *p* = .020, *BF*_*10*_ = 8.29). The (pre-)adolescents however, showed negative values for the distractor repetition cost in both predictability conditions (low: −8.20 ± 2.55; high: −11.20 ± 4.21), which did not significantly differ from one another (*p* = .569, *BF*_*10*_ = −4.05).

Lastly, the third ANOVA also revealed a significant three-way interaction of trial type*predictability*group (*F*(1,66) = 4.452, *p* = .039, *η*_*p*_^*2*^ = .063, *BF*_*10*_ = 18.19). According to the Bonferroni-corrected post-hoc tests, RT_pro_ of RCDC trials did not differ between predictability conditions (*M*_*Diff*_ = 1.80 ms ± 3.96, *p* = .651, *BF*_*10*_ = −8.22), but between groups, with adults being significantly faster than (pre-)adolescents (*M*_*Diff*_ = 95.62 ms ± 16.16, *p* < .001, *BF*_*10*_ = 25.01). Similarly, adults were significantly faster in the RCDR trials in general (*M*_*Diff*_ = 101.00 ms ± 15.99, *p* < .001, *BF*_*10*_ > 100). Our previous paper (Jamous et al., 2024) already showed that adults significantly improved their RT_pro_ of RCDR trials in the high predictability condition (*M*_*Diff*_ = −10.55 ms ± 4.82, *p* = .036, *BF*_*10*_ *=* 1.50) up to the same performance as RCDC trials. In contrast, RT_pro_ of RCDR trials were not affected by predictability for the (pre-)adolescents (*M*_*Diff*_ = 2.91 ms ± 5.68, *p* = .610, *BF*_*10*_ *=* 0.96).

In summary, (pre-)adolescents did not benefit from the high predictability condition as adults do: While healthy adults seem to be able to decrease binding costs through high predictability by improving their performance in conditions where only task-irrelevant features are repeated, no effect of predictability on any of the behavioral performance measures was observed for healthy (pre-)adolescents.

### 3.2 Time-frequency analysis

Time-frequency transformation was performed for theta (4–7 Hz) and alpha (8–12 Hz) band activity to compare oscillatory activity in different predictability conditions within the (pre-)adolescent group and between groups. As in our previous work, the within-group difference was calculated by subtracting RCDR high minus RCDR low predictable condition. Within the adult sample, cluster-based permutation testing revealed significant differences in TBA after the prime stimulus and in ABA around the probe stimulus when comparing partial-overlap distractor repetition trials of high and low predictability conditions (Jamous et al., 2024). The behavioral results suggest that (pre-)adolescents do not show the oscillatory modulation around the probe stimulus (i.e., during retrieval) that was revealed for adults (Jamous et al., 2024). Yet, it is unclear if they show oscillatory modulation after the prime stimulus (i.e., during binding), similar to the adults.

**Fig. 4:**
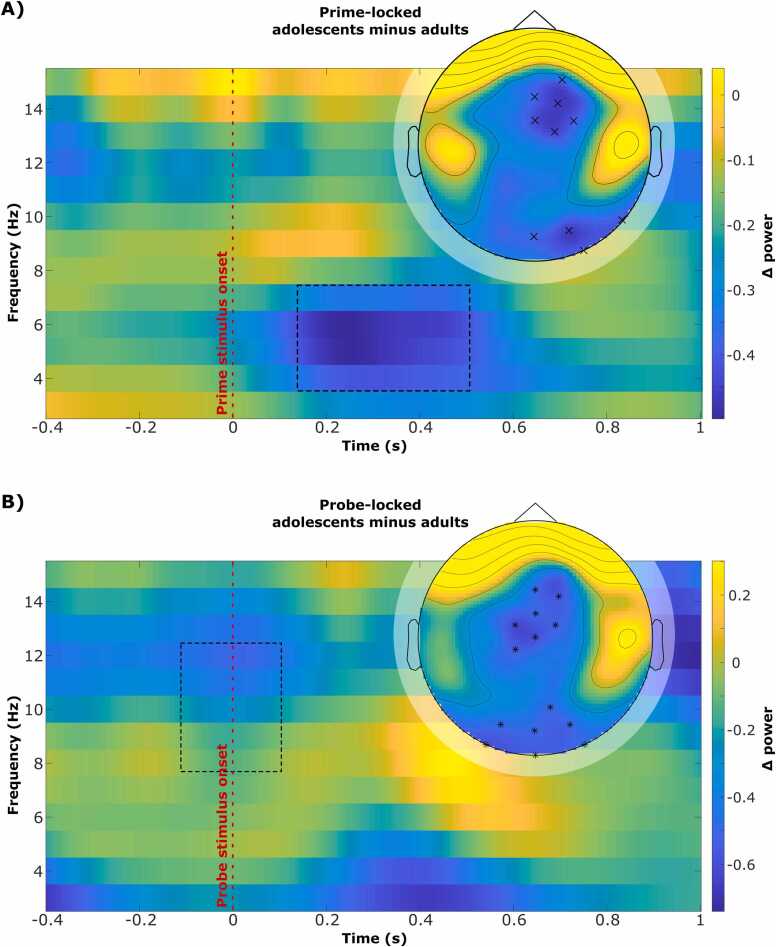
Group differences in prime- and probe-locked time-frequency transformation. Time-frequency transformation and topoplot of significant negative clusters for the group difference (adolescents minus adults) in high predictability RCDR trials. The displayed power is averaged **(baseline normalized using decibel conversion)** over the electrodes of the significant clusters while the boxes with dashed lines represent the corresponding time window: A): 150–500 ms after prime stimulus onset and B) −100–100 ms around probe stimulus onset.

In contrast to the results within the adult sample, no significant clusters were found in either ABA or TBA within the (pre-)adolescent sample for the comparison of high and low predictability conditions. To cross-validate these findings, oscillatory activities were compared between the groups ((pre-)adolescents minus adults) within each predictability condition: There were no significant ABA or TBA clusters between (pre-)adolescents and adults in the low predictability condition (prime- and probe-locked). In the high predictability condition, two negative clusters in TBA (adults > (pre-)adolescents) were found 150–500 ms after prime stimulus onset at fronto-central (*p* = .016) and occipital electrodes (*p* = .030; see Fig. 4**A**). Similarly, two negative clusters in ABA (adults > (pre-)adolescents) were revealed around probe stimulus onset (-100–100 ms) at fronto-central (*p* = .003) and occipital electrodes (*p* = .005; see Fig. 4**B**).

In alignment with the behavioral results, (pre-)adolescents and adults did not differ in the low predictability condition, but the high predictability condition, with adults showing higher TBA after the prime and higher ABA around the probe. Thus, (pre-)adolescents do not show distinct oscillatory modulations for high and low predictability within the trials that were identified as crucial for the performance improvement in adults.

### 3.3 MVPA results

Classifications were applied around both prime stimulus onset and probe stimulus onset (-1000–1000 ms) within each group.

#### 3.3.1 MVPA around prime stimulus onset

**Fig. 5:**
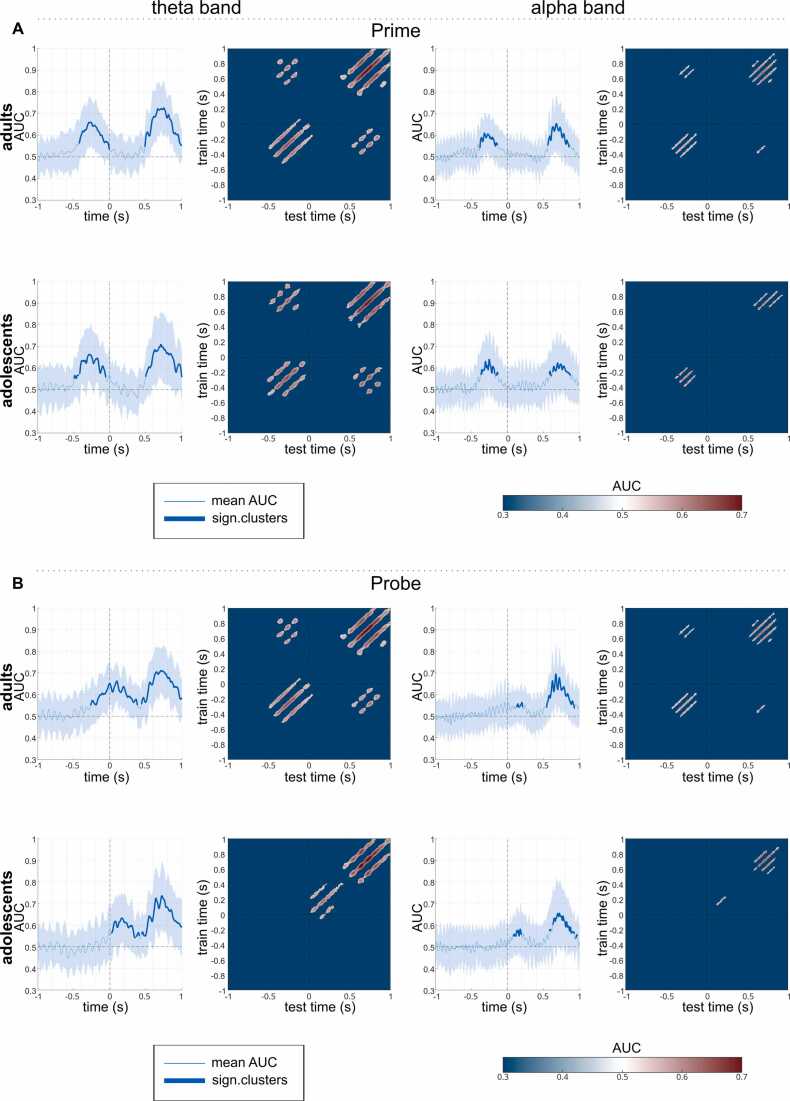
*Temporal MVPA results.* Temporal MVPA results within the adult and (pre-)adolescent sample around the A) prime stimulus onset (time point 0) using theta band training and prediction sets (left side) and alpha band prediction set (right side), and around the B) probe stimulus onset (time point 0). For each group and condition, plots of the AUC curve over time are displayed on the left, with the thicker line indicating when and for how long the classification was significantly above the chance level and the shaded area displaying the standard deviation. Temporal generalization plots are displayed right next to it.

Using theta-band training and prediction sets, the MVPA on the sensor-level theta power time course and subsequent cluster-based permutation testing revealed that the MVPA was able to classify the high and low predictability trials significantly above chance level for both groups (see Fig. 5**A**). For the adults, the AUC was significant in the time window of approx. 400–0 ms before prime stimulus onset (AUC_mean_ = 0.61, AUC_max_ = 0.66, AUC_min_ = 0.53) as well as in the time window of approx. 500–1000 ms after prime stimulus onset (AUC_mean_ = 0.65, AUC_max_ = 0.73, AUC_min_ = 0.55). The AUC of the (pre-)adolescents was significant in almost identical time-windows - however, the first time window started already approx. at 500 ms and lasted until 80 ms before the prime stimulus onset (AUC_mean_ = 0.61, AUC_max_ = 0.66, AUC_min_ = 0.55) while the second time window was found again approx. 500–1000 ms after prime stimulus onset (AUC_mean_ = 0.65, AUC_max_ = 0.71, AUC_min_ = 0.56). Similarly, using alpha-band training and prediction sets, the MVPA on the sensor-level alpha power time course and subsequent cluster-based permutation testing revealed that the MVPA was able to differentiate between the high and low predictability conditions significantly above chance level for both groups (see Fig. 5**A**). The AUC of the adult sample was significant in the time window of approx. 400–150 ms before prime stimulus onset (AUC_mean_ = 0.58, AUC_max_ = 0.61, AUC_min_ = 0.54) as well as in the time window of approx. 550–850 ms after prime stimulus onset (AUC_mean_ = 0.60, AUC_max_ = 0.65, AUC_min_ = 0.54). For the (pre-)adolescents, the AUC was significant in the time window of approx. 350–150 ms before prime stimulus onset (AUC_mean_ = 0.60, AUC_max_ = 0.64, AUC_min_ = 0.57) as well as in the time window of approx. 580–880 ms after prime stimulus onset (AUC_mean_ = 0.59, AUC_max_ = 0.62, AUC_min_ = 0.56).

#### 3.3.2 MVPA around probe stimulus onset

Using theta-band training and prediction sets, the MVPA on the sensor-level theta power time course and subsequent cluster-based permutation testing revealed that the MVPA was able to classify the high and low predictability trials significantly above chance level for both groups (see Fig. 5**B**). The AUC of the adult sample was significant in the time window of approx. 250 ms before until 1000 ms after the probe stimulus onset (with a small break between 390 and 450 ms after onset, AUC_mean_ = 0.62, AUC_max_ = 0.71, AUC_min_ = 0.55). For the (pre-)adolescents, the AUC was significant in the time window of approx. 20–1000 ms before prime stimulus onset (with a small break between 400 and 450 ms after onset, AUC_mean_ = 0.62, AUC_max_ = 0.74, AUC_min_ = 0.54). Similarly, using alpha-band training and prediction sets, the MVPA on the sensor-level alpha power time course and subsequent cluster-based permutation testing revealed that the MVPA was able to differentiate between the high and low predictability conditions significantly above chance level for both groups (see Fig. 5**B**). For the adults, the AUC was significant in the time window of approx. 100–200 ms (AUC_mean_ = 0.55, AUC_max_ = 0.56, AUC_min_ = 0.54) as well as in the time window of approx. 550–900 ms after probe stimulus onset (AUC_mean_ = 0.61, AUC_max_ = 0.70, AUC_min_ = 0.54). The AUC of the (pre-)adolescents was significant in almost identical time-windows with the first time window of approx. 80–200 ms before probe stimulus onset (AUC_mean_ = 0.56, AUC_max_ = 0.58, AUC_min_ = 0.55), and the second time window approx. starting only 600 ms after the probe stimulus onset and lasting until 900 ms (AUC_mean_ = 0.60, AUC_max_ = 0.66, AUC_min_ = 0.54).

The MVPA results for adults and adolescents are very similar – both around the prime and the probe stimulus in each, theta and alpha frequency band. In particular, the classifier performs significantly above chance level even before the prime stimulus, i.e., regardless of trial type and visual influences. This suggests that both groups have distinct internal representations of the two predictability conditions, reflecting a mature ability to represent predictability cues. Nevertheless, the subsequent exploitation of this distinct internal representations to their benefit (i.e., more efficient integration and retrieval of perception-action features by suppressing task-irrelevant features) is lacking.

## 4 Discussion

The present study investigated whether (pre-)adolescents are able to use information about the probability of task-irrelevant features to mitigate the negative effects of perception-action integration (binding costs; e.g., Colzato et al., 2006; Dilcher et al., 2021a; Hommel, 1998, Hommel, 2004; Moeller and Frings, 2014; Singh et al., 2018). Prior research has shown that adults can implicitly exploit high predictability of task-irrelevant features to shield attention and improve performance, especially under conditions that require the flexible retrieval or suppression of event files (Jamous et al., 2024, Schmalbrock et al., 2022). We hypothesized that if children and adolescents can similarly internalize predictability information, they might show reduced binding costs, reflecting enhanced attentional control and more efficient perception-action integration. In any case, this provides further insights into the maturation of attentional and action control.

The behavioral findings revealed that (pre-)adolescents did not benefit from high predictability in the way adults do. Binding effects remained stable across conditions, with neither costs nor benefits significantly modulated by predictability. This suggests that, unlike adults, (pre-)adolescents did not utilize the probabilistic structure of task-irrelevant features to enhance action control. These findings align with previous evidence indicating that attentional control – especially the ability to suppress irrelevant information – continues to mature throughout adolescence (Cowan et al., 2006, Hobbiss and Lavie, 2024, Huang-Pollock et al., 2002).

Importantly, the neurophysiological data further supported these behavioral findings. In adults, high predictability has been linked to ABA modulations around the probe stimulus, thought to reflect attentional shielding and more efficient retrieval of relevant information (Jamous et al., 2024). In the current study, however, (pre-)adolescents did not show comparable ABA modulations, indicating the absence of this shielding effect during retrieval. Moreover, no TBA modulation was observed after the prime stimulus – another pattern robustly linked to adults’ integration of predictability information into the event file itself. In our previous paper, we proposed that TBA modulation reflects increased binding of predictability information, which may serve as a foundation for efficient retrieval later on (Jamous et al., 2024). The absence of both ABA and TBA modulations in our (pre-)adolescent sample suggests that these participants neither integrated predictability information into the event-file nor used them to guide retrieval. In the light of Lenartowicz et al. (2025) and Foster and Awh (2019), the present findings may also suggest that alpha modulations represent a flexible adjustment of cortical excitability and prioritization rather than pure suppression. In this sense, ABA could serve as a dynamic gate that coordinates when sensory information enters action‑relevant event files. Similarly, TBA may index both the detection of contextual change and the coupling of distributed control networks that implement adjustments to predicted events. The absence of these coordinated alpha-theta modulations in (pre‑)adolescents thus reflects not only weaker inhibitory control but also immature integration of excitability‑based signaling into top‑down control loops.

This raises a critical question: Were (pre-)adolescents unable to connect this information to the other perception-action features, or did they simply fail to notice the difference in predictability? Judging by previous research, children are able to learn implicitly at an early age and apply what they have learned directly (e.g., Amso and Davidow, 2012; Lukasova et al., 2018; Meulemans et al., 1998; Reber, 2013; Vinter and Perruchet, 2000). This would indicate that participants were able to recognize the pattern creating predictability. To explore this, we applied a temporal generalization MVPA to neural activity around prime and probe onset, respectively, isolating the mental representation of the high versus low predictability condition. Interestingly, the classifier performed significantly above chance in both age groups, indicating that the two conditions were indeed represented differently in the brain, even in (pre-)adolescents. Crucially, these distinct internal representations of the two conditions are already found before the prime, i.e., the first stimulus. On the one hand, this suggests that (pre-)adolescents were not entirely insensitive to the differing predictability degrees. On the other hand, their failure to benefit from high predictability as evidence by the on a behavioral and neurophysiological data implies that this internal distinction did not translate into task-relevant modulation. It is possible that the classifier captured neural differences unrelated to predictability per se, such as block structure or general attentional fluctuations. Yet, the participants may have formed abstract contextual representations without linking them to distractor dynamics or binding demands. Therefore, future studies should explicitly manipulate participants’ awareness of the predictability to determine whether (pre-)adolescents can leverage this information when it is clearly available. Another potential explanation is developmental in nature: Even though implicit learning mechanisms are known to be largely evident in children and adolescents, their limited attentional control (Munakata et al., 2012) may constrain their ability to proactively apply learned regularities immediately (Chevalier et al., 2015) to shield attention (Wang and Theeuwes, 2018) and flexibly update event-files. That is, while the capacity to form internal representations of predictability may be present, the executive mechanisms required to use this information efficiently might still be underdeveloped. Another potential explanation is computational in nature. From a predictive-processing perspective, predictability must be weighted against sensory precision and current control goals (Friston, 2010). This weighting process – mediated by prefrontal control and theta/alpha band activity coupling (Cavanagh and Frank, 2014) – depends on mature top-down modulation. If these mechanisms are still developing, adolescents may encode regularities but fail to increase their precision weighting, leaving prediction signals behaviorally inert. The current results thus converge with the idea that while implicit learning mechanisms are largely established early in life, the neural systems that dynamically integrate these learned regularities into control policies continue to mature across adolescence.

It is important to note that the current findings must be interpreted in light of the high interindividual variability within the (pre-)adolescent sample. Unlike adults, developmental populations are characterized by substantial heterogeneity, with even small age differences resulting in meaningful cognitive and neurophysiological variability (e.g., Bodmer et al., 2018; De Ribaupierre and Lecerf, 2018; Dilcher et al., 2021b; Genon et al., 2022). Future research should consider narrower age bands or developmental subgroups to more precisely characterize when and how sensitivity to predictability emerges and whether it can be facilitated through explicit instruction or training.

In summary, while children and adolescents are capable of implicit learning and seem to form distinct internal representations, they did not benefit from high predictability in reducing binding-related costs in the current task. This inability to harness predictability information behaviorally or neurophysiologically suggests a developmental gap in the flexible control of perception-action integration. Although (pre-)adolescents should be able to represent different environmental contexts, they appear unable to integrate such information into event-files or use it to support attentional shielding. These findings highlight a critical dissociation between context representation and task-relevant control in developing action systems and suggest that the ability to modulate cognitive control based on probabilistic structure may be a hallmark of mature action control.

## Data statement

Data from the study can be obtained from the corresponding author upon reasonable request.

## CRediT authorship contribution statement

**Roula Jamous:** Writing – review & editing, Writing – original draft, Visualization, Investigation, Formal analysis, Data curation, Conceptualization. **Leonore Luise Ilgenstein:** Investigation, Data curation. **Katharina Graf:** Writing – review & editing, Writing – original draft, Visualization, Methodology, Investigation, Formal analysis, Conceptualization. **Christian Frings:** Writing – review & editing, Resources, Funding acquisition, Conceptualization. **Christian Beste:** Writing – review & editing, Writing – original draft, Supervision, Resources, Project administration, Funding acquisition, Conceptualization.

## Declaration of Competing Interest

The authors declare no conflict of interest

## Data Availability

Data will be made available on request.
